# First Insight into a Nationwide Genotypic Diversity of* Mycobacterium tuberculosis* among Previously Treated Pulmonary Tuberculosis Cases in Benin, West Africa

**DOI:** 10.1155/2017/3276240

**Published:** 2017-06-21

**Authors:** Dissou Affolabi, N'Dira Sanoussi, Sergio Codo, Fréderic Sogbo, Prudence Wachinou, Faridath Massou, Aderemi Kehinde, Séverin Anagonou

**Affiliations:** ^1^Faculty of Health Sciences, Abomey-Calavi University, Cotonou, Benin; ^2^National Reference Laboratory for Mycobacteria, Cotonou, Benin; ^3^Department of Medical Microbiology & Parasitology, College of Medicine, University of Ibadan, Ibadan, Nigeria; ^4^Department of Medical Microbiology & Parasitology, University College Hospital, Ibadan, Nigeria

## Abstract

**Background:**

Molecular studies on tuberculosis (TB) are rare in low-resource countries like Benin, where data on molecular study on previously treated TB cases is unavailable.

**Materials and Methods:**

From January to December 2014, all smear- and culture-positive previously treated pulmonary TB patients from all TB clinics were systematically recruited. Drug susceptibility testing and spoligotyping were performed on all isolates.

**Results:**

Of the 100 patients recruited, 71 (71.0%) were relapse cases and 24 (24.0%) were failure cases, while 5 (5.0%) were default cases. Resistance rate to any first-line drug was 40.0%, while 12.0% of strains were multidrug-resistant (MDR) and no strain was extensively drug-resistant (XDR). A total of 40 distinct spoligotypes were found to be corresponding to a genotypic diversity of 40.0%. ST61 was the most predominant spoligotype with prevalence of 33.0%. In all, 31 single spoligotypes and nine clusters were observed with 2 to 33 strains per cluster giving a clustering rate of 69.0%. Euro-American (Lineage 4) was the most prevalent lineage (74.0%) and Lineage 2 was associated with resistance to streptomycin.

**Conclusion:**

This first insight into genetic diversity of previously treated pulmonary TB patients in Benin showed a relatively high genetic diversity of* Mycobacterium tuberculosis*.

## 1. Introduction

Tuberculosis (TB) remains a global public health problem. According to World Health Organization (WHO), an estimated number of 10.4 million new cases occurred in the world in 2015 [1]. The African Region recorded the highest incidence rate, almost twice that of the world [1]. In Benin in West Africa, 4,092 cases were detected in 2015 [2].

Despite use of standardized treatment regimens and a well-established National TB Program (NTP) in the country, the treatment success rate as well as the number of previously treated cases (failure, relapse, and default) has remained stable over years [2]. In contrast to new cases, previously treated cases are much more likely to harbour multidrug-resistant (MDR) strains, defined as resistance to rifampicin (R) and isoniazid (H), and their characteristics may differ from those of new cases [3, 4].

Molecular tools are useful for better understanding of TB transmission dynamics in a given area. Nevertheless, molecular studies on TB are scarce in high-incidence, low-income countries [5]. In Benin, the only molecular epidemiologic study available to our knowledge recruited only TB new cases in one city [6, 7]. The scarcity of these studies in TB endemic countries is partly due to lack of resources and relative complexity of some molecular techniques. Among them, spoligotyping has the advantage of being relatively simple, inexpensive, and generally sufficient as a first approach of molecular epidemiology of TB [8].

In this study, we aimed to evaluate a nationwide genotypic diversity of* Mycobacterium tuberculosis* complex strains in previously treated pulmonary TB patients in Benin, using spoligotyping technique.

## 2. Materials and Methods

### 2.1. Setting

Benin is a country with a size of 114,763 square kilometres and an estimated population of 11 million. It has 70 TB facilities spread all over the country and a well-established National TB Program. Every year, about 4,000 TB cases including new and previously treated cases are detected in the country [2].

### 2.2. Specimens

A total of 100 isolates obtained from 100 sputum samples collected from smear-positive previously treated pulmonary TB patients all over the country were sent to the National Reference Laboratory (NRL) in Cotonou for processing. Previously treated TB patients were from relapse (*n* = 71), failure (*n* = 24), and default (*n* = 5) cases. Two sputum samples were collected (spot and early morning) from each patient, stored at 4°C, and sent in a cool box to the NRL within a week. Upon arrival at the NRL, the two samples were processed for culture but only one strain per patient was used for drug susceptibility testing (DST) and DNA fingerprinting. Samples were systematically collected between January and December 2014, and, for each of them, demographic data was retrieved, while after obtaining consent from each patient, HIV screening was performed on blood using rapid immunochromatography-based tests: Alere Determine HIV-1/2® (Alere Medical, Japan) was used for HIV screening, while samples that were reactive were confirmed by ImmunoComb HIV 1&2 BiSpot® (Orgenics, France).

### 2.3. Culture and DST

Samples were decontaminated using the Petroff method and cultured on Löwenstein-Jensen (LJ) media [9]. The* M. tuberculosis* isolates (one per patient) were tested for susceptibility against rifampicin (R), isoniazid (H), streptomycin (S), and ethambutol (E) using the proportion method on LJ medium at the following concentrations: 40 *μ*g/mL, 0.2 *μ*g/mL, 4 *μ*g/mL, and 2 *μ*g/mL, respectively [9]. Internal quality control was routinely performed, while annual external quality assurance was carried out by the WHO Supranational Reference Laboratory at the Institute of Tropical Medicine, Antwerp, Belgium. In case of resistance to R, DST for second-line drugs was performed using the proportion method on LJ medium at the following concentrations: kanamycin (30 *μ*g/mL), capreomycin (40 *μ*g/mL), amikacin (40 *μ*g/mL), and ofloxacin (2 *μ*g/mL) [10]. All strains were stored upon routine processing at −80°C and subcultured on LJ for spoligotyping.

### 2.4. Spoligotyping

DNA was extracted by making a suspension of bacteria with a loop of colonies into 300 *μ*L of molecular grade water followed by heating at 100°C for 20 minutes. Spoligotyping was performed as previously described [11].* Mycobacterium tuberculosis* H37Rv was used as a positive control, while molecular grade water served as negative control. Spoligotype patterns obtained were then translated into binary code with 1 and 0 for presence and absence of “spacer” and then entered on an Excel file. From these codes, lineages and families of strains were determined using TB lineage database http://tbinsight.cs.rpi.edu/run_tb_lineage.html [12] and the SPOTCLUST database http://tbinsight.cs.rpi.edu/run_spotclust.html [13], respectively. Spoligotype data were compared to the SITVIT WEB database (http://www.pasteur-guadeloupe.fr:8081/SITVIT_ONLINE/) [14] to determine the Spoligotype International Type (SIT) if already described.

### 2.5. Data Analysis

Data were analyzed using EpiData 3.1. Chi-square test and Fisher's exact test were used to compare proportions.* p* value < 0.05 was considered significant.

## 3. Results

In total, 100 viable strains (one single strain per patient) were used for spoligotyping. They were 71, 24, and 5 isolates from relapse, failure, and default patients, respectively. In total, 74 (74.0%) isolates were from male patients, while 26 (26.0%) were from females. HIV positivity rate was 15.2%, all of whom were infected with HIV1.

Resistance pattern to first-line drugs by type of previously treated cases is presented in Table 1. Resistance rate to any first-line drug was 40.0%, while 12.0% of strains were multidrug-resistant (MDR). In addition, two other strains were resistant to R but not to H; one was monoresistant to R and another one was resistant to both R and S. Thus, resistance rate to R was 14.0%. MDR rates were 20.8% and 9.9% for failure and relapse cases, respectively, while none was found among defaulters. Second-line DST results were available for nine MDR strains, of which six (66.7%) were susceptible, two (22.2%) showed resistance to ofloxacin, and one (11.1%) showed resistance to kanamycin, while none was resistant to both fluoroquinolones and injectable drugs. Thus, no strain was extensively drug-resistant (XDR) (Table 2).

A total of 40 distinct spoligotypes were found to be corresponding to a genotypic diversity of 40.0%. Of these, 21 (52.5%) corresponded to spoligotypes already identified in the SITVIT database and had shared-type (ST) denominations (SIT), while 19 (47.5%) were newly found spoligotypes. ST61, ST53, and ST1 were the most predominant spoligotypes with prevalence rates of 33.0%, 13.0%, and 8.0%, respectively. In this study, 31 single spoligotypes and nine clusters were observed with 2 to 33 strains per cluster, giving a rate of 69.0% (Table 3).

Most prevalent families were LAM 10, T1, and* M. africanum* West-African 1 with prevalence rates of 46.0%, 17.0%, and 12.0%, respectively. For lineages, the more prevalent lineages were Euro-American (Lineage 4),* M. africanum* West-African 1 (Lineage 5), and East-Asian (Lineage 2), with prevalence rates of 74.0%, 12.0%, and 8.0%, respectively. Interestingly, one strain was identified as* M. bovis*, representing 1.0% of the total strains tested (Table 4).

By comparing characteristics of patients within lineages, we found no association between sex, HIV status, types of treatment, and lineages; however, drug resistance particularly resistance to S was associated with lineages distribution. Strains belonging to Lineage 2 were more likely to be resistant to S than the other strains (*p* = 0.001) (Table 5).

## 4. Discussion

There are still several gaps in understanding TB dynamics in Africa. For example, the reason why* M. africanum* is mainly restricted to the Western and Central parts of the continent remains unclear [5, 7]. Studies using molecular tools may be useful in this respect. Unfortunately, the few molecular studies available either were limited to a city or a region or only focused on new TB cases and if previously treated cases were included, the number was usually low [15, 16].

In this study, we carried out a nationwide molecular study on previously treated pulmonary TB cases detected in Benin over a period of one year. In total, 40 different spoligotypes were found, corresponding to a genotypic diversity of 40.0%. This percentage was higher than the 19.1% found by Ouassa et al. in previously treated cases in Côte d'Ivoire but was quite similar to 35.1% obtained on the genetic diversity in a mixed population of new and previously treated cases in Rwanda [15, 16]. A genotypic diversity of 49.0% was reported in 2005 among new cases in Cotonou, the biggest city in Benin, suggesting that genetic diversities were similar among new and previously treated cases [6]. However, the previous study among new cases was carried out 10 years ago and distribution of spoligotypes in new cases might have changed over time. In addition, the national figure might be different from what was obtained in Cotonou.

This study showed that the most frequent spoligotype was ST61 (33%) belonging to the Latino-American and Mediterranean (LAM) family. This finding was similar to what was previously reported in the same country in 2005, indicating that ST61 was the most prevalent spoligotype in new cases [6]. This same genotype was previously described to be prevalent in countries within the West-African coast [17].

At a lineage level, Lineage 4 was the most prevalent lineage (74.0%). High prevalence of Lineage 4 was also found in both new and previously treated cases at a similar rate in Ethiopia (72.4%) and in Guinea (78.8%) [18, 19]. In comparison with other lineages, Lineage 4 appears to have certain characteristics that promote its rapid expansion. For* M. bovis*, the prevalence rate (1.0%) is similar to those found elsewhere in a mixed population of new and previously treated cases in Ethiopia (1.2%), Nigeria (1.0%), and Mali (0.8%) [20–22]. These low proportions could be explained by the fact that* M. bovis* is usually involved in extrapulmonary TB in humans, whereas most of these studies, including the present one, were on pulmonary TB [20–22].

A significant association was found between Lineage 2 (Beijing strains) and resistance to streptomycin (*p* = 0.001). This same association was observed in a study of new cases in 2005 in Cotonou, where an outbreak characterised by Information Geographical System was identified [23]. The positive correlation between Lineage 2 and resistance to streptomycin suggests a clonal distribution of Beijing strains in Benin.

This study showed that, in Benin, molecular signatures of strains causing TB retreatment cases are similar to those causing new cases. The reasons why these strains did not respond to first-line TB treatment are likely to be related to human and environmental factors rather than the intrinsic molecular characteristics of strains. Therefore, for TB control, national TB programs in Benin as well as in neighbouring countries should make efforts to reduce the impact of these factors in order to decrease the number of TB retreatment cases.

In this study, the resistance rate to R was 14.0%. This rate was slightly higher than what was observed by Affolabi et al. in Benin in 2013 (10%) but was comparable to that of the national drug resistance survey in 2010 [24, 25]. Furthermore, this rate was similar to what was reported by Homolka et al. among previously treated cases in Sierra Leone (14.4%) but was lower than 43.7% reported by Dia et al. in Senegal [26, 27]. Among MDR cases, 33.3% were pre-XDR but no XDR strain was found, contrary to findings from Burkina Faso, Ethiopia, and many other countries in Sub-Saharan Africa [28–30]. This absence of XDR strains in this study could be explained by the rigorous management of the MDR-TB program in Benin with strict application of directly observed therapy during the whole nine-month treatment course. However, the threat of XDR-TB is always present with the emergence of pre-XDR cases and a need for more vigilance cannot be overemphasized.

In conclusion, this first insight into the genetic diversity of TB in previously treated cases in Benin showed a genetic diversity of 40.0%, with most strains belonging to Lineage 4, similar to previous data in new TB cases. Occurrence of retreatment cases is more likely to be related to human and environmental factors rather than the intrinsic molecular characteristics of strains.

## Figures and Tables

**Table 1:** Resistance pattern of strains to first-line drugs.

Type of resistance	Failure (*n* = 24) *n* (%)	Relapse (*n* = 71) *n* (%)	Default (*n* = 5) *n* (%)	Total (*n* = 100) *n* (%)
Susceptible to all drugs	12 (50.0)	45 (63.4)	3 (60.0)	60 (60.0)
Monoresistance				
H	1 (4.2)	1 (1.4)	0 (0.0)	2 (2.0)
S	3 (12.5)	10 (14.1)	1 (20.0)	14 (14.0)
R	0 (0.0)	0 (0.0)	1 (20.0)	1 (1.0)
E	1 (4.2)	1 (1.4)	0 (0.0)	2 (2.0)
Total	5 (20.8)	12 (16.9)	2 (40.0)	19 (19.0)
Multidrug resistance				
HR	0 (0.0)	2 (2.8)	0 (0.0)	2 (2.0)
HRE	1 (4.2)	1 (1.4)	0 (0.0)	2 (2.0)
HRS	1 (4.2)	1 (1.4)	0 (0.0)	2 (2.0)
HRES	3 (12.5)	3 (4.2)	0 (0.0)	6 (6.0)
Total	5 (20.8)	7 (9.9)	0 (0.0)	12 (12.0)
Other patterns				
HS	1 (4.2)	1 (1.4)	0 (0.0)	2 (2.0)
HSE	0 (0.0)	2 (2.8)	0 (0.0)	2 (2.0)
RS	0 (0.0)	1 (1.4)	0 (0.0)	1 (1.0)
ES	1 (4.2)	3 (4.2)	0 (0.0)	4 (4.0)
Total	2 (8.3)	7 (9.9)	0 (0.0)	9 (9.0)

H: isoniazid; E: ethambutol; S: streptomycin; R: rifampicin.

**Table 2:** Resistance patterns to second-line drugs on MDR strains.

Type of resistance	MDR strains *n* = 9 *n* (%)
Susceptible to all second-line drugs	6 (66.7)
Monoresistance	
Ofloxacin	2 (22.2)
Kanamycin	1 (11.1)
Capreomycin	0
Amikacin	0
Total	*3 (33.3)*
XDR	0

XDR: extensively drug-resistant.

**Table 3:** Strains per family.

Family	Spoligotype	ST	Strains *n* (%)
Family33	761777767775771	U	1 (1.0%)
	777777777763771	54	1 (1.0%)

Family34	777777770000000	46	1 (1.0%)

Beijing	000000000003771	1	8 (8.0%)

CAS	703777740001171	1199	1 (1.0%)

LAM1	677777607760771	20	1 (1.0%)

LAM9	377777607760771	177	1 (1.0%)

LAM10	777777743760771	61	33 (33.0%)
	767740741760751	U	1 (1.0%)
	777770343760771	U	1 (1.0%)
	777677743760771	U	3 (3.0%)
	777770343740771	U	2 (2.0%)
	777777743460771	772	3 (3.0%)
	777777742760771	U	1 (1.0%)
	777777743760731	403	1 (1.0%)
	777777743740771	U	1 (1.0%)

T1	777777777760771	53	13 (13.0%)
	777777777760731	51	1 (1.0%)
	737777777760731	848	1 (1.0%)
	777777757760771	44	1 (1.0%)
	737777777760531	U	1 (1.0%)

T2	777417707700000	U	1 (1.0%)

T4	777740017760771	159	1 (1.0%)

Haarlem1 (H1)	777777770020731	316	1 (1.0%)

Haarlem2 (H2)	000000000020731	U	1 (1.0%)

Haarlem3 (H3)	777777777720731	49	2 (2.0%)
	777777777720771	50	2 (2.0%)

Family36	000000007760771	4	1 (1.0%)

*M. africanum *West-African 1	774077607777071	331	3 (3.0%)
	674077717777071	U	1 (1.0%)
	774077400603031	U	1 (1.0%)
	770002607777071	U	1 (1.0%)
	574077607777071	319	1 (1.0%)
	774077600000071	U	1 (1.0%)
	374077607777031	U	1 (1.0%)
	574017607777071	U	1 (1.0%)
	774040077777071	U	1 (1.0%)
	774077777777071	438	1 (1.0%)

*M. africanum *West-African 2	700000377777671	U	1 (1.0%)

*M. bovis*	000040000200000	U	1 (1.0%)

ST: shared-type; U: unknown.

**Table 4:** Strains per lineage.

Lineage	Denomination	*n*	%
1	Indo-Oceanic	3	3.0
2	East-Asian	8	8.0
3	East-African-Indian	1	1.0
4	Euro-American	74	74.0
5	*M. africanum* West-African 1	12	12.0
6	*M. africanum* West-African 2	1	1.0
*M. bovis*	*M. bovis*	1	1.0
Total		*100*	*100.0 *

**Table 5:** Association between lineages and resistance.

Lineage	Resistance to S *n* (%)	No resistance to S *n* (%)
1	1 (33.3)	2 (66.7)
2	7 (87.5)	1 (12.5)
3	1 (100.0)	0 (0.0)
4	17 (23.0)	57 (77.0)
5	4 (40.0)	6 (60.0)
6	0 (0.0)	1 (100.0)
*M. bovis*	1 (100.0)	0 (0.0)
Total	*31 (31.6)*	*67 (68.4)*

S: streptomycin.

## Abbreviations

DST: Drug susceptibility test
E: Ethambutol
H: Isoniazid
LJ: Löwenstein-Jensen
LAM: Latino-American and Mediterranean
MDR: Multidrug-resistant
NRL: National Reference Laboratory
R: Rifampicin
SIT: Spoligotype International Type
S: Streptomycin
ST: Shared-type
TB: Tuberculosis
WHO: World Health Organization
XDR: Extensively drug-resistant.

## References

[1] (2016). *Global Tuberculosis Report*.

[2] (2015). *Rapport Annuel*.

[3] Caminero J. A. (2010). Multidrug-resistant tuberculosis: epidemiology, risk factors and case finding. *International Journal of Tuberculosis and Lung Disease*.

[4] Rockwood N., Abdullahi L. H., Wilkinson R. J., Meintjes G. (2015). Risk factors for acquired rifamycin and isoniazid resistance: A systematic review and meta-analysis. *PLoS ONE*.

[5] de Jong B. C., Antonio M., Gagneux S. (2010). Mycobacterium africanum-review of an important cause of human tuberculosis in West Africa. *PLoS Neglected Tropical Diseases*.

[6] Affolabi D., Anyo G., Faïhun F., Sanoussi N., Shamputa I. C., Rigouts L. (2009). First molecular epidemiological study of tuberculosis in Benin. *International Journal of Tuberculosis and Lung Disease*.

[7] Gehre F., Antonio M., Faïhun F. (2013). The First Phylogeographic Population Structure and Analysis of Transmission Dynamics of M. africanum West African 1- Combining Molecular Data from Benin, Nigeria and Sierra Leone. *PLoS ONE*.

[8] Ei P. W., Aung W. W., Lee J. S., Choi G.-E., Chang C. L. (2016). Molecular strain typing of Mycobacterium tuberculosis: A review of frequently used methods. *Journal of Korean Medical Science*.

[9] (1998). *Laboratory Services in Tuberculosis Control. Part III*.

[10] World Health Organization, 2012 http://www.stoptb.org/wg/gli/assets/documents/Updated%20critical%20concentration%20table_1st%20and%202nd%20line%20drugs.pdf

[11] Kamerbeek J., Schouls L., Kolk A., Van Agterveld M., Van Soolingen D., Kuijper S. (1997). Simultaneous detection and strain differentiation of *Mycobacterium tuberculosis* for diagnosis and epidemiology. *Journal of Clinical Microbiology*.

[12] Shabbeer A., Cowan L. S., Ozcaglar C. http://Tbinsight.cs.rpi.edu/about_tblineage.html.

[13] Vitol I., Driscoll J., Kreiswirth B., Kurepina N., Bennett K. P. (2006). Identifying *Mycobacterium tuberculosis* complex strain families using spoligotypes. *Infection, Genetics and Evolution*.

[14] Demay C., Liens B., Burguière T. (2012). SITVITWEB—a publicly available international multimarker database for studying *Mycobacterium tuberculosis* genetic diversity and molecular epidemiology. *Infection, Genetics and Evolution*.

[15] Ouassa T., Borroni E., Loukou G. Y. (2012). High Prevalence of Shared International Type 53 among Mycobacterium tuberculosis Complex Strains in Retreated Patients from Côte d'Ivoire. *PLoS ONE*.

[16] Gafirita J., Umubyeyi A. N., Asiimwe B. B. (2012). A first insight into the genotypic diversity of Mycobacterium tuberculosis from Rwanda. *BMC Clinical Pathology*.

[17] Godreuil S., Torrea G., Terru D. (2007). First molecular epidemiology study of Mycobacterium tuberculosis in Burkina Faso. *Journal of Clinical Microbiology*.

[18] Garedew L., Mihret A., Mamo G. (2013). Strain diversity of mycobacteria isolated from pulmonary tuberculosis patients at Debre Birhan Hospital, Ethiopia. *International Journal of Tuberculosis and Lung Disease*.

[19] Ejo M., Gehre F., Barry M. D. (2015). First insights into circulating Mycobacterium tuberculosis complex lineages and drug resistance in Guinea. *Infection, Genetics and Evolution*.

[20] Traore B., Diarra B., Dembele B. P. P. (2012). Molecular strain typing of Mycobacterium tuberculosis complex in Bamako, Mali. *International Journal of Tuberculosis and Lung Disease*.

[21] Lawson L., Zhang J., Gomgnimbou M. K. (2012). A molecular epidemiological and genetic diversity study of tuberculosis in Ibadan, Nnewi and Abuja, Nigeria. *PLoS ONE*.

[22] Nuru A., Mamo G., Worku A. (2015). Genetic Diversity of Mycobacterium tuberculosis Complex Isolated from Tuberculosis Patients in Bahir Dar City and Its Surroundings, Northwest Ethiopia. *BioMed Research International*.

[23] Affolabi D., Faïhun F., Sanoussi N. (2009). Possible outbreak of streptomycin-resistant Mycobacterium tuberculosis Beijing in Benin. *Emerging Infectious Diseases*.

[24] Affolabi D., Faihun F., Migeo K G., Adè S., Adè G., Makpenon A. Anti-tuberculosis drug resistance among new and previously treated pulmonary tuberculosis patients in Benin.

[25] Ade S., Adjibodé O., Wachinou P., Toundoh N., Awanou B., Agodokpessi G. (2016). Characteristics and treatment outcomes of retreatment tuberculosis patients in Benin. *Tuberculosis Research and Treatment*.

[26] Homolka S., Post E., Oberhauser B. (2008). High genetic diversity among Mycobacterium tuberculosis complex strains from Sierra Leone. *BMC Microbiology*.

[27] Dia M. L., Sow A. I., Cisse M. F., Gueye P., Ba F., Cisse N. N. (2016). Resistance profile of mycobacteria isolated from patients undergoing retreatment in Senegal. *The Journal of Infectious Diseases*.

[28] Agonafir M., Lemma E., Wolde-Meskel D., Goshu S., Santhanam A., Girmachew F. (2010). Phenotypic and genotypic analysis of multidrug-resistant tuberculosis in Ethiopia. *International Journal of Tuberculosis and Lung Disease*.

[29] Saleri N., Badoum G., Ouedraogo M. (2010). Extensively drug-resistant tuberculosis, Burkina Faso. *Emerging Infectious Diseases*.

[30] Gehre F., Otu J., Kendall L. (2016). The emerging threat of pre-extensively drug-resistant tuberculosis in West Africa: Preparing for large-scale tuberculosis research and drug resistance surveillance. *BMC Medicine*.

